# Editorial: Investigating emotional expressions and coping in sport from a sociocultural perspective

**DOI:** 10.3389/fpsyg.2024.1390548

**Published:** 2024-03-13

**Authors:** Jiajin Yuan, John Elvis Hagan, Xianxin Meng

**Affiliations:** ^1^Sichuan Key Laboratory of Psychology and Behavior of Discipline Inspection and Supervision (SICNU), Institute of Brain and Psychological Sciences, Sichuan Normal University, Chengdu, China; ^2^Department of Health, Physical Education and Recreation, University of Cape Coast, Cape Coast, Ghana; ^3^School of Psychology, Fujian Normal University, Fuzhou, China

**Keywords:** sociocultural perspectives, emotion regulation, stress coping, sports psychology, mental health, subjective wellbeing, depression, emotional experience

The experience, responding and effective coping of emotion or stress play a vital role in humans' adaptive life in the changing environments (Chen et al., 2020; Yang et al., 2022). From the everyday understanding of social intentions and mindsets, to the management of stress and intense emotions individuals encounter during challenging situations, the maintenance of external interpersonal relationships and internal mental health all rely on the appropriate and functional coping strategies (Yang et al., 2012; Chen et al., 2017, 2020; Wang et al., 2023). In empirical studies, evidence shows that proper performance of sports not only entails the involvement of emotion regulation and stress coping, particularly in competitive situations, the sports itself also serves as an important avenue to emotion regulation/stress coping (Shen et al., 2023).

The desirable display of sports competence cannot be realized without emotion/stress regulation. There is empirical evidence showing that the strategic use of mental training from high-level athletes optimize precompetitive psychosocial and bodily states related to emotional stress, which in turn contribute to the improvements of competition performance (Robazza et al., 2004). Conversely, the self-depletion of emotion regulatory resources, such as improper use of expressive suppression, significantly hinders actual competitive performance like completing a 10 km cycling task slower, generating lower mean power outputs, and reaching a lower maximum heart rate (Wagstaff, 2014). Further, physical sports itself serves as an important avenue of emotional management. For example, sports like mindful movement of Tai Chi Chuan combined with aerobic exercise and meditation, have proven effective in the intervention of daily emotional disturbances, hypervigilance and anxiety, as well as depressive symptoms. These goals, are attained through the neural hub of the prefrontal cortex and interoceptive sensitivity (Yao et al., 2021). Also, long-term sports training has a stable, sustainable function of reducing anxiety levels, overall negative emotions, and their corresponding physiological indicators like improved heart-rate variability (Shen et al., 2023).

Despite the mutual interactions between sports activity and mental health measures, exploring the dynamic relationships between the two from the lens of sociocultural differences across the nations, ethnics and subcultural groups are still scarce. To complement this area, we organized this topic with the aim of broadening the current understanding of the sports-mental health dynamics from sociocultural perspectives. The current topic, to date, has gathered four empirical studies related to sports psychology in terms of stress or emotional management and sports-assisted mental health maintenance. Thereafter, a brief introduction of them has been done as follows according to the time of publication:

First, Ding et al. investigated the influence of religion on subjective wellbeing. Specifically, the authors used the 2018 data of Taiwan Social Change Survey (TSCS) using moderated mediation models to examine the association between religious type and subjective wellbeing. Though the results did not show a significant correlation between the type of religious belief and subjective wellbeing, respondents who adhere to institutional religion inclusive of Islam, Christianity and Catholicism have a higher frequency of participating in religious activities, which serves as a protective factor in the improvement of subjective wellbeing. Moreover, cross-regional comparisons showed a pattern of “empty-cup effect” (those with less baseline value benefited more from the proposed intervention), in that religious believers in rural areas had a significantly higher wellbeing than those in urban areas, while those living in urban areas were more inclined to participate in religious activities frequently to gain an enhanced sense of wellbeing. Despite lack of direct relevance for emotion-coping in sports, these data provided some incremental evidence for the understanding of the relation between religious belief and wellbeing and of social-cultural profiles across different Taiwanese regions in stress coping and the maintenance of mental health.

Agormedah et al. set out their fundamental study based on a prevalent phenomenon of using religious coping strategy in athletes due to the stressful experiences before and during games, such as coping with uncertain sporting outcomes. Therefore, in order to investigate the assessment validity of sports-related religious coping, the authors tested the reproducibility of the brief religious coping instrument (RCOPE) with three-hundred African athletes, across three African countries with an aim to test sociocultural invariance and cross-gender stability for the inventory. The key results illustrated a two-factor dimension construct, positive religious coping and negative religious coping, with all items for either dimension contributing significantly to the measure of the instrument. Further, the use of the instrument for athletes' religious coping has desirable validity for both males and females, and for respondents from each of three countries (Benin, Ghana, Nigeria).

Mulvenna et al. also tested the relationship between self-based goals itself with underlying motivational reasons, and sports-related emotions (pride and shame) in a UK park-runner sample, using the methodology of structural equation modeling for data analyses. The study focused on confirming whether challenge or threat appraisal of stress would account for the influence of self-based goals and underlying motivations on running performance and corresponding emotional consequences. Key findings in this three-wave longitudinal study indicate that self-approach goals, as characterized by orientation to better accomplishment anticipation relative to a past record, leads to more challenge appraisal and less threat appraisal of the forthcoming 5 km parkrun activity which, in turn, contributed to the enhancement of pride following the parkrun sports. In addition, pursing a self-avoidance goal such as avoiding doing worse this time compared to the performance of last time undermined

parkrun time and contributed to the feeling of shame post event. This line of findings highlights the importance of appraising the sports as a resource-inspiring, opportunity and growth-related challenge instead of self-diminishing threats in the explanation of the association between self-determined goals and post-sports emotional experiences.

The last study by Mansell and Turner explored the profiles of how individuals appraise the nature of stress influences people's psychological wellbeing assessed through vitality and depressive symptoms, and how these experiences differ as a function of sports expertise between athletes and non-athletes. Specifically, the authors' primary focus was on the role that proactive coping plays in the relationship between stress mindset and challenge appraisal tendencies and further tested how this relationship modulated psychological wellbeing. Key findings highlighted the importance of possessing an enhancing stress mindset (i.e., adopting a “stress is enhancing” instead of “stress-is-debilitating”) perspective. This perspective is suggestive of a proactive, growth-oriented coping and appraisal of stress as challenge for the promotion of vitality and reduction of depressive symptoms. Specifically, the positive relationship between stress mindset and challenge appraisal tendencies was observed and this relationship is mediated by proactive coping. Also, challenge appraisal tendencies were positively associated with vitality, which were negatively associated with depressive symptoms. Concerning the comparisons between athletes and non-athletes, the authors observed that athletes reported a significantly greater “stress-is-enhancing” mindset, greater vitality, and fewer depressive symptoms than non-athletes, implying a potential role of sports training in the cultivation of optimistic cognitive construct and adaptive stress coping. These results provided support for the role that stress mindset has in influencing psychological wellbeing and depicted how the appraisal of stress and proactive coping contributed to adaptive mental health outcomes.

As aforementioned, these four studies explored the emotion/stress coping and its association with sports from different sociocultural perspectives. However, in terms of the theme investigating emotional expression and coping in sports, there remain several issues that should be noted as future directions, especially those unresolved under the current topic. Firstly, most of the included papers for the current topic paid the primary emphasis on the ways or mechanisms of maintaining mental health and adaptive behavior, in combination of exploration into sociocultural moderators of mental health outcomes. However, the roles of emotional understanding, recognition and emotional expression in sports were seldomly assessed. Recent studies have highlighted the important role of interpersonal emotion regulation, built on emotional understanding/recognition in performing team sports (Campo et al., 2017), while there is evidence that emotional expressions of coaches have significant influences on players' performance, in that coaches' expressions of happiness but not anger were conducive to team performance in sports (van Kleef et al., 2019). In this sense, future studies should pay more attention to mutual influences between emotional understanding/expression and sports performances. Secondly, the current analyses of sociocultural influences on mental health and sports outcomes primarily focused on different nations and subregions. To better understand how sociocultural factors moderate the outcomes of interest, research should pay more attention to setting up or distinguishing sociocultural variables such as different ethnic groups, different types of religion believers, likewise to classic studies of cultural differences in emotion regulation using an approach of discriminating interdependent, east-Asian from independent, north American self-values (Butler et al., 2007). Setting up specific sociocultural variables in combination with a suitable control of relevant demographic variables, also helps to tell where the observed group differences may come from and of which cultural difference represents. Similarly, the choice of this approach also provides an opportunity to determine what the cross-cultural group similarity or lack of group differences mean. Thirdly, the current collection of papers all used observational approaches, either cross-sectional or longitudinal. Experimental causal studies are still necessary in order to elucidate the mechanisms behind the protective pathways to mental health outcomes in sports.

## Author contributions

JY: Conceptualization, Funding acquisition, Investigation, Resources, Supervision, Writing – original draft, Writing – review & editing. JH: Conceptualization, Resources, Supervision, Writing – original draft, Writing – review & editing. XM: Conceptualization, Investigation, Resources, Validation, Writing – original draft, Writing – review & editing.
